# Aberrant Non-Coding RNA Expression in Patients with Systemic Lupus Erythematosus: Consequences for Immune Dysfunctions and Tissue Damage

**DOI:** 10.3390/biom10121641

**Published:** 2020-12-06

**Authors:** Chang-Youh Tsai, Chieh-Yu Shen, Chih-Wei Liu, Song-Chou Hsieh, Hsien-Tzung Liao, Ko-Jen Li, Cheng-Shiun Lu, Hui-Ting Lee, Cheng-Sung Lin, Cheng-Han Wu, Yu-Min Kuo, Chia-Li Yu

**Affiliations:** 1 Division of Allergy, Immunology & Rheumatology, Taipei Veterans General Hospital and National Yang-Ming University, Taipei 11217, Taiwan; cwliu2@vghtpe.gov.tw (C.-W.L.); darryliao@yahoo.com.tw (H.-T.L.); 2 Division of Rheumatology, Immunology, & Allergy, Department of Internal Medicine, National Taiwan University Hospital, Taipei 10002, Taiwan; tsichhl@gmail.com (C.-Y.S.); hsiehsc@ntu.edu.tw (S.-C.H.); dtmed170@yahoo.com.tw (K.-J.L.); b89401085@ntu.edu.tw (C.-S.L.); chenghanwu@ntu.edu.tw (C.-H.W.); 543goole@gmail.com (Y.-M.K.); 3 Institute of Clinical Medicine, National Taiwan University School of Medicine, Taipei 10002, Taiwan; 4 Mackay Memorial Hospital and Mackay College of Medicine, Taipei 10449, Taiwan; htlee1228@gmail.com; 5 Department of Thoracic Surgery, Ministry of Health and Welfare Taipei Hospital, New Taipei City 24213, Taiwan; doc2765c@ms59.hinet.net

**Keywords:** SLE, ncRNA, miRNA, lncRNA, circRNA, epigenetic regulation, autoimmunity

## Abstract

Systemic lupus erythematosus (SLE) is a complex systemic autoimmune disease with heterogeneous clinical manifestations. A diverse innate and adaptive immune dysregulation is involved in the immunopathogenesis of SLE. The dysregulation of immune-related cells may derive from the intricate interactions among genetic, epigenetic, environmental, and immunological factors. Of these contributing factors, non-coding RNAs (ncRNAs), including microRNAs (miRNAs, miRs), and long non-coding RNAs (lncRNAs) play critical roles in the post-transcriptional mRNA expression of cytokines, chemokines, and growth factors, which are essential for immune modulation. In the present review, we emphasize the roles of ncRNA expression in the immune-related cells and cell-free plasma, urine, and tissues contributing to the immunopathogenesis and tissue damage in SLE. In addition, the circular RNAs (circRNA) and their post-translational regulation of protein synthesis in SLE are also briefly described. We wish these critical reviews would be useful in the search for biomarkers/biosignatures and novel therapeutic strategies for SLE patients in the future.

## 1. Introduction

Systemic lupus erythematosus (SLE) is a prototype of chronic systemic autoimmune disease, characterized by the presence of diverse autoantibodies against different cellular, nuclear, and extracellular components with consequent chronic inflammation and tissue damage. Factors that are implicated in lupus pathogenesis include genetic/epigenetic predisposition, environmental stimulants, sex hormone imbalance, mental/psychological stresses, and certain undefined conjectural factors. These diverse contributing parameters may lead to complex immune dysfunctions in patients with SLE via the breakdown of self-tolerance. Dysregulations in innate [1,2,3,4,5,6,7,8,9,10] and adaptive [11,12,13,14,15] immune systems have been recently widely recognized. The innate immune cells include neutrophils [4,16], macrophages/dendritic cells [1,2,3,17] and natural killer cells [5,6,18,19]. The adaptive immune cells include T lymphocyte subpopulations, Th1, Th2, Th17, Treg, CD45 RO^+^ memory T (T_m_) and follicular helper T (T_fh_) cells [11,12,20,21,22,23,24], as well as B lymphocytes, CD5^+^ B cells, and regulatory B (B_reg_) cells [13,14,15,25,26,27]. A comprehensive review on the immunopathogenesis of SLE has been newly given [28]. In addition, aberrant intracellular signaling in the immune-related cells relevant to lupus pathogenesis has also been recently explored, including suppressor of cytokine signaling (SOCS) [29,30], signal transducers and activators of transcription (STATs) [31], type 1 interferon (IFN-1) [32,33], and ubiquitin, which regulate IFN-α receptor expression [34]. In our previous investigations, Li et al. [35] have reported that deranged bioenergetics and defective redox capacity in T cells and neutrophils are relevant to defective cellular immunity in SLE patients. Lee et al. [36,37,38,39] have further demonstrated that leukocyte mitochondrial DNA alterations and dysfunctions can increase oxidative stresses and are closely related to lupus pathogenesis. Thus, our group has proposed that increased oxidative and nitrosative stresses may enhance immunosenescence, inflammation, and “inflamm-aging” in patients with SLE [40]. Table 1 summarizes the abnormal cell biology in the immune-related cells together with their aberrant intracellular signaling and immunometabolism that are relevant to lupus pathogenesis. Apparently, the immunological dysfunctions are the final outcome of the patients with SLE originated from the up-stream genetic/epigenetic regulation for controlling the disease development. In the subsequent sections, we first discuss the genetic predisposition based on the big data derived from human genome-wide association studies (GWAS). Then, the sophisticated epigenetic regulations implicated in lupus pathogenesis were further divided into three parts: (1) regulation of pre-transcriptional gene expression by DNA methylation/acetylation and histone modifications, and the post-translational non-histone protein modifications; (2) modulation of the post-transcriptional mRNA expression by intracellular ncRNAs; and (3) modulation of the post-transcriptional mRNA expressions by extracellular cell-free ncRNAs. These scenarios are successively discussed in detail in the following sections.

## 2. Understanding the Genetics/Epigenetics of Patients with SLE in the Post-GWAS Era

In the past decades, using the data from GWAS, many authors have tried to explore the complicated genetic structures of SLE. Large-scale genetic association studies have uncovered a substantial fraction of the genomic heritability of SLE [41,42,43,44]. In combination with recently developed next-generation sequencing (NGS) studies, authors have identified over 100 genetic loci in association with SLE [45,46]. However, nearly all identified variants are located within the non-coding regions [46,47,48]. In particular, the variants are enriched within super-enhancers, stretch enhancers, and multiple-enhancers [49]. More interestingly, a significant proportion of risk alleles demonstrated in the GWAS play essential roles in B cell activation [48,50] and signaling relevant to the induction of IFN-1s [51]. Accordingly, the pathogenesis of SLE can be further accounted for by the big data with regard to its molecular basis [52,53].

### 2.1. Genetic Loci Susceptible to Lupus Pathogenesis

It is conceivable that pathological autoantibodies and immune complexes (ICs) are responsible for diverse tissue inflammation and damage via complement activation in patients with SLE. In the course of IC formation, some of these autoantibodies per se and the ICs may activate complements and result in deleterious effects. Normally, the Fcγ receptors and complement receptors are crucial for clearing these noxious molecules for preventing the accompanying IC deposition and inflammatory reactions. Defects in IC clearance-related components may result in the development of lupus. Fielder et al. [54] have found that the presence of null alleles for C2, C4A, and C4B are relevant to genetic susceptibility to SLE. Furthermore, immunoglobulin Fc receptors are key components for amplification of chronic inflammation in SLE [55]. Genetic variants of Fc receptors have been found associated with SLE susceptibility and disease severity [56,57]. Low copy numbers of *FCGR3B* are a risk factor for SLE [58,59]. The classical major histocompatibility complex (MHC) genes, i.e., human leukocyte antigen (HLA), are associated with ordinary immune function. In the HLA regions, class II genes are dominant as SLE susceptibility loci, including HLA-DRB1 (DRB1*1501 and DRB1*0301) [60,61]. In addition to the above IC processing and HLA-associated genetic loci, many genes are related to the immune signal transduction. For example, Toll-like receptor (TLR)/IFN-1 and other pathways are relevant to lupus pathogenesis [43,44,45,46,47,48,49,50,51,52,53]. Besides, genes specific for B cell activation and signaling implicated in the induction of IFN-1 have also been discovered [46,47,50,51]. The HLA and non-HLA associated loci involved in lupus pathogenesis in the post-GWAS era are listed in Table 2.

### 2.2. Epigenetic Regulation in Patients with SLE

Epigenetics is the study of heritable changes in gene functions that occur without a change in genetic codes. The basis of epigenetic regulation of gene expression includes DNA methylation/acetylation, pre-transcriptional gene expression with chromatin remodeling by histone modifications, gene transcription by post-translational non-histone protein modifications, and intracellular and cell-free ncRNAs for post-transcriptional interference for mRNA expression [62]. The ncRNAs are arbitrarily divided into microRNAs (miR with 20–24 nucleotides) and long non-coding RNAs (lncRNAs with >300 nucleotides). These four parts are discussed in detail in the following subsections.

#### 2.2.1. DNA Hypomethylation in SLE-CD4^+^ T Cells

DNA methylation is a biochemical process involving the addition of a methyl moiety to a cytosine or adenine residue at repeated CpG dinucleotides (CpG island) in gene promoter regions for repressing gene expression, which is part of the epigenetic regulation process of gene expression (Table 3) [63,64,65,66,67,68,69,70,71,72,73,74]. The biochemical process is mediated by DNA methyl-transferase (DNMT) 1, 3a, and 3b. On the contrary, gene transcription restoration by demethylation of the promoter CpG islands is achieved by ten-eleven translocation (TET) enzymes TET1, TET2, and TET3. The genome-wide methylation pattern has been studied by many authors and has revealed that in addition to classical methylation-sensitive related genes in SLE-CD4^+^ T cells including CD11a (*ITGAL*), perforin (*PRF1*), CD70 (*TNFSF7*), CD40 ligand (*TNFSF5*) and *PP2Ac*α, reported by Zhang et al. [63], the tyrosine kinase gene (*TNK2*), the phosphatase gene (*DUSP5*) and type I IFN master regulator gene (*IRF7*) are also involved as reported by Coit et al. [64]. Imgenberg-Kreuz et al. [65] further demonstrated that CD45 (*PTPRC*), MHC-class II, HLA-DQB2, *UHRFBP1*, *IRF5*, *IRF7*, *IKZF3,* and *UBE2L3* are also associated with SLE pathogenesis. Yeung et al. [66] have found that hypomethylation of genes related to type I IFN pathway including *MX1*, *IF144L*, *NLRC5,* and *PLSCR* are also involved in these events, which solidify the importance of IFN-α in lupus pathogenesis [67]. In clinical regards, Weeding et al. [68] have found that hypomethylation of *IF144L* is highly sensitive and highly specific for SLE. The hypomethylation of transcriptional factor enhancer of Zeste homolog 2 (EZH2) plays an important role in triggering SLE disease activity. Joseph et al. [69] have reiterated that nine IFN-related genes, including *MX*, *IFI44L*, *PARP9*, *DT3XL*, *IFIT1*, *IFI44*, *RSAD_2_*, *PLSCR_1,_* and *IRF_7_* are implicated in type I IFN pathway activation, and thus are relevant to the disease activity of SLE (SLEDAI). Coit et al. [64] have even reported that *CG10152449* in *CHST_12_* hypomethylation in CD4^+^ T cells has a very high specificity (64.3%) to the patients with lupus nephritis. De la Calle-Fabregat [70] concluded that DNA methylation is essential for immune differentiation, and its derangement is highly implicated in the development of this autoimmune disease. Zhang et al. [71] found that *IFI*35 hypomethylation in CD3^+^ T cells can enhance the proliferation of mesangial cells relevant to lupus nephritis by deleting the methylation status of lupus kidneys.

#### 2.2.2. DNA Hydroxymethylation by miRNA Interference in Patients with SLE

5-hydroxymethylcytosine (5-hmc) is the oxidative derivative of 5-methylcytosine (5-mc) that is generated by TET1, 2, and 3. The conversion of 5-mc to 5-hmc is associated with DNA demethylation to facilitate effector T cell differentiation [72]. Sui et al. [73], by genome-wide analysis of 5-hmc in peripheral blood of SLE patients, found that 5-hmc levels of *TREX1, CDKN1A,* and *CDKN1B* are significantly enhanced in SLE. Furthermore, Zhao et al. [74] analyzed SLE-CD4^+^ T cells and identified increased *SOCS1*, *NR2F6,* and *IL15RA* DNA hydroxymethylation, contributing to lupus pathogenesis.

#### 2.2.3. Alternation of Histone Modifications in Immune-Related Cells in SLE

Gene expression is also controlled by chromatin tightness via complex mechanisms where the structural changes in histones are one of the mechanisms. The histone modification may include methylation acetylation, citrullination, phosphorylation, ubiquitination, and SUMOylation, among which methylation and acetylation are mostly investigated. Histone acetylation transferase (HAT) and histone deacetylase (HDAC) can effectively catalyze the addition or removal of acetyl moiety on the lysine (K) residue of histones. Acetylation relaxes the chromatin structure by diminishing the electric charge between histone and DNA. In contrast, deacetylation tightens the chromatin structure to silence the gene expression.

Hu et al. [75] detected the global histone H3/H4 acetylation and H3K4/H3K9 methylation in SLE-CD4^+^ T cells. They found the global histone H3 and H4 hypoacetylation in active lupus-CD4^+^ T cells, whereas the global H3K4 methylation was not different between patients and controls. In addition, the histone H3 lysine 4 trimethylation (H3K4me3) variants in SLE-peripheral blood mononuclear cells (PBMCs) were found with significant alteration of H3K4me3 structure associated with lupus pathogenesis [76]. Zhang et al. [77] demonstrated that H4 acetylation was significantly altered in monocytes of SLE patients, which is potentially regulated by IRF1. Zhou et al. [78] have demonstrated again that histone H3 acetylation and H3K4me levels in CD4^+^ T cells were significantly elevated and positively correlated with disease activity in SLE. They concluded that aberrant histone modifications in *TNDSF7* (CD70) promotor contribute to the development of SLE by increasing CD70 expression in CD4^+^ T cells.

T cells from SLE patients exhibit permissive histone modifications at the IL-17 gene cluster by increasing H3K18ac and decreasing H3K27me that presumably lead to overexpression of pro-inflammatory IL-17A [78,79,80,81]. Moreover, the silencing of the IL-2 gene is caused by impairing histone H3K18 deacetylation and H3K27me3 with an increase in methylation [78,79,80,81]. As for the immune regulatory and anti-inflammatory IL-10 gene, authors have demonstrated that H3K18ac (acetylation) is responsible for its increased expression in SLE [79,80]. Another pro-inflammatory cytokine, TNF-α, has been reported to be increased in expression as associated with H3ac in SLE-monocytes [79,80,81]. The increase in H3K4me3 for IRF1 binding site was also reported in SLE-monocytes [82].

#### 2.2.4. Post-Translational Non-Histone Protein Modifications in SLE

Post-translational modifications (PTMs) of proteins are defined as covalent modifications at a specific amino acid residue in protein in a timely and signaling manner. This biochemical event can happen in the peripheral tissues, such as at inflammation sites, or in the thymus to modify a specific protein antigenicity. Consequently, the modified self-antigens can be taken as foreign molecules and processed by antigen-presenting cells (APCs) to evoke autoantibody production and/or autoreactive T cells. The PTMs found in patients with SLE include phosphorylation, methylation, acetylation, isoaspartylation, etc. The proteins susceptible to phosphorylation in SLE are U1 small nuclear ribonucleoprotein (snRNP) 68K [83], SSA/Ro and SSB/La [84,85], spliceosomal Sm protein that is vulnerable to methylation in D1 and D3 subunits [86,87], the frequently ubiquitinated proteinase serine/arginine-rich splicing factor 1 (SKSF1) [88] and myeloperoxidase (MPO) [89,90].

Table 4 lists the histone and non-histone protein modifications in immune-related cells and post-translational molecules in SLE patients.

### 2.3. Aberrant Expression of ncRNAs in Patients with SLE

The most recently discovered epigenetic mechanisms for gene expression are dependent on ncRNAs. These non-translatable small nucleotides, including microRNAs (miRs, with 20–24 bp in size) and long non-coding RNAs (lncRNAs <300 bp in size), were initially regarded as housekeeping molecules. However, these ncRNAs not only act as post-transcriptional regulators for mRNA expression but can interact mutually between themselves (miRNAs and lncRNAs) to further modulate the epigenetic, transcriptional, translational, and peptide localization modifications [91,92]. In addition to the intracellular ncRNAs, more and more aberrant extracellular cell-free ncRNA expression in plasma, saliva, urine, or tissues has been identified as associated with autoimmune and inflammatory diseases [93,94].

#### 2.3.1. Aberrant Intracellular ncRNA Expression Associated with Pathogenesis and as Biomarkers/Biosignatures in SLE Patients

Fan et al. [95] identified that microRNA (miR)-31 is a novel enhancer for IL-2 production during T cell activation, and decreased expression of miR-31 is now considered to be a unique molecular mechanism underlying IL-2 deficiency in SLE. Lu et al. [96] demonstrated that under-expressed miR-145 and over-expressed miR-224 accelerate T cells to undergo activation-induced cell death (AICD) in SLE since STAT1 mRNA is targeted by miR-145 and apoptosis inhibitory protein 5 (AIP5) is targeted by miR-224. These aberrant enhancement events would be associated with lupus nephritis by stimulating STAT1 expression in SLE-T cells. The same group further demonstrated that Ca^++^ influx-regulated miRNAs, miR-524-5p and miR-449b, are overexpressed in SLE-T cells. These findings may explain the enhanced INF-γ production and are in parallel with SLE disease activity (SLEDAI) [97]. Khoshmirsafa et al. [98] proved that miR-21 and miR-155 levels in PBMC are significantly greater in patients with active lupus nephritis and can be used as biomarkers for SLE patients.

**Figure 1:**
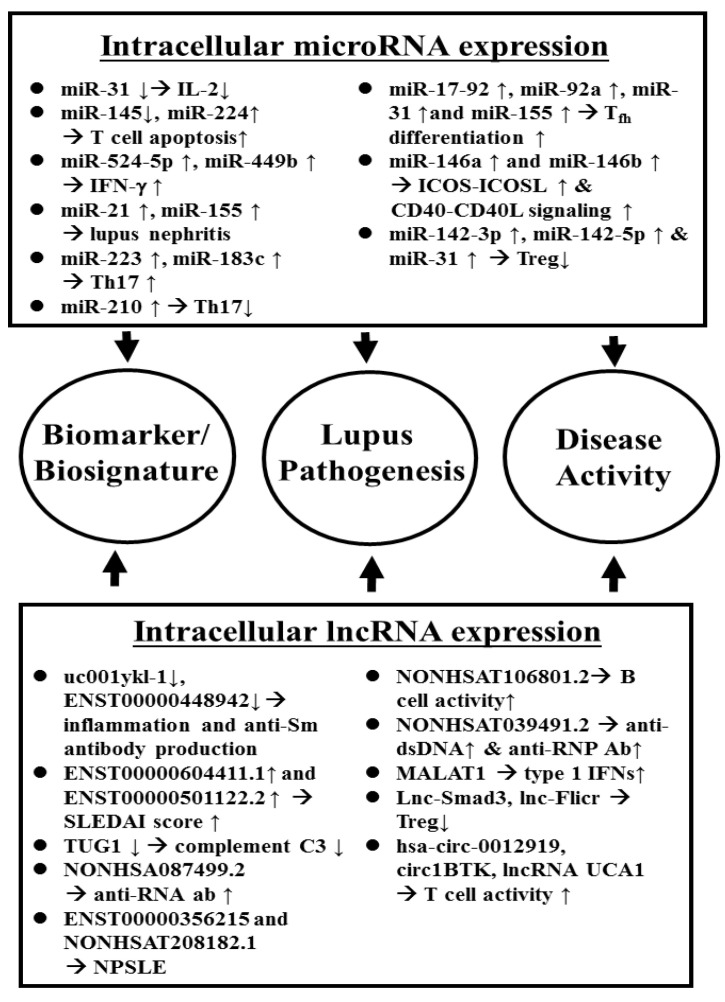
Aberrant intracellular ncRNA expression in the pathogenesis, and as disease biomarkers/biosignatures as well as disease activity index of SLE.

On the other hand, it is believed that lncRNAs mediate “sponge-like” effects on different miRs and consequently suppress miR-mediated activities. Li et al. [99] showed that downregulated uc001ykl.1 and ENST00000448942 in SLE-CD3^+^ T cells. The expression of uc001ykl.1 is correlated with ESR and CRP, whereas ENST00000448942 level is correlated with ESR and anti-Sm antibodies. Wang et al. [100] demonstrated that lncRNAs, ENST00000604411.1, and ENST00000501122.2, were upregulated, and lncRNAs, lnc-HSFY2-33, and lnc-SERPINB9-1:2, were down-regulated in SLE-monocytes/dendritic cells. They concluded that the expression levels of ENST00000604411-1 and ENST00000501122.2 were positively correlated with SLEDAI scores, respectively. Cao et al. [101] found that low complement C3 levels were positively correlated with decreased *TUG1* expression in PBMCs in SLE with nephritis. Geng et al. [102] revealed that NONHSA087499.2 level correlated with anti-RNA antibody and ENST00000356215 level correlated with olfactory thresholds and oral ulcers. NONHSAT208182.1 correlated with the presence of fever or unstable gait. NONHSAT106801.2 correlated with B cell and fever. NONHSAT024353.2 correlated with serum IgG level and the presence of anti-SSA. NONHSAT039491.2 was associated with lupus activity and the presence of anti-dsDNA, anti-RNP, and other neuropsychiatric manifestations. Gao et al. [103] have found that *MALTAT1* is involved in type 1 IFN-mediated SLE by up-regulating *OAS2*, *OAS3*, and *OASL*, confirming further the report by Ye et al. [104] claiming that full high-throughput sequencing analysis of lncRNA expression profile in SLE-PBMCs could reveal a robust “IFN signature”. A comprehensive review of ncRNA in SLE-CD4^+^ T cells with new insights into lupus pathogenesis has been published by Gao et al. [105]. Figure 1 depicts the involvement of intracellular ncRNAs in the pathogenesis, use as biomarkers/biosignatures, and disease activity monitoring indicators in patients with SLE.

#### 2.3.2. Abnormal Cell-Free ncRNA Expression as Biomarkers/Biosignatures and in the Pathogenesis of SLE

The extracellular vesicles (EVs) carry nucleic acids, proteins, and lipids, and play essential roles in many intercellular communications and intracellular functions. The size of EV ranges from 30 nm to 5 μm, including exosomes of 30–100 nm, microvesicles/microparticles of 100–1000 nm, and apoptotic bodies of ~5000 nm. The nucleic acids contained in EVs may include DNAs, mRNAs, and ncRNAs [106]. These small lipid-bilayered spherical vesicles are released by different kinds of cells and can be found in different biofluids such as plasma, urine, saliva, CSF, synovial fluid, and breast milk [107]. When EVs are transferred to remote recipient cells, the carried-on epigenetic signals can be transferred as important commands in cell-cell communications [108]. Recent investigations have revealed that the EV-derived ncRNAs and cell-free lncRNAs in the body fluid play crucial roles in autoimmune and inflammatory diseases and thus may not only serve as biomarkers but as therapeutic agents or targets, especially for SLE [106,109]. In this review, we focus on the aberrant expression of cell-free ncRNAs in the plasma, urine, and nephritic tissues of SLE patients in the following discussion.

#### 2.3.3. An Abnormal Cell-Free ncRNA Expression in SLE

Wang et al. [110] firstly reported that serum and urinary cell-free miR-146a and miR-155 participated in the pathophysiology and could serve as biomarkers for SLE. Carlsen et al. [111] demonstrated that seven circulating miRs are involved in the pathogenesis of SLE in which miR-142-30 and miR-181a are increased, but miR-106a, miR-17, miR-20a, miR-203, and miR-92a are decreased in the plasma. In addition, the expression of miR-342-3p, miR-223, and miR-20a was significantly decreased in SLE patients with active nephritis. Among these seven miRs, four can target transforming growth factor (TGF)-β1 signaling pathways, and the other three modulate the regulation of cell apoptosis, interactions between cytokine receptors, T cell development, and cytoskeletal organization. Furthermore, Wang et al. [112] demonstrated that the up-regulation of serum miR-130b-3p could negatively influence 3′-UTR of ERBB2IP to suppress its expression and play an important role in the early stages of lupus nephritis.

Amr et al. [113] have attempted to investigate the regulatory biomarkers in T cell activation in SLE patients. They found that miR-31 was expressed lower while miR-21 is expressed higher in SLE, suggesting a significant association between miR-21/miR-31 balance and their impact on tuning the IL-2 pathway of T cell activation in SLE. Kay et al. [114] reported three decreased cell-free circulatory miRs, miR-125b, miR-101, and miR-375, which were indicative of atherosclerosis.

For diagnostic purposes, authors have reported a number of circulating miRs as novel biomarkers associated with clinical parameters. Kim et al. [115] showed that hsa-miR-30e-5p, hsa-miR-92a-3p, and hsa-miR-223-3p were significantly up-regulated and present in SLE plasma, among which hsa-miR-223-3p was significantly related to oral ulcers and lupus anti-coagulant. Zeng et al. [116] confirmed that elevated miR-271b-5p and miR-5100 expression in SLE serum could be used as biomarkers associated with clinical parameters. In addition, Navarro-Quiroz et al. [117], by analyzing profiles of miRs in peripheral blood from patients with class IV lupus nephritis, identified 14 miRs to be associated with lupus nephritis. Zhang et al. [118] confirmed that 14 B cell-related circulating cell-free miRs (miR-103, miR-150, miR-20, miR-223, miR-27a, miR-15b, miR-16, miR-181a, miR-19b, miR-22, miR-23a, miR-25, miR-92a, and miR-93) are significantly decreased in SLE-plasma. Furthermore, the down-regulated expression of miR-19b, miR-25, miR-93, and miR-15b is associated with lupus disease activity. The lower expression of miR-15b and miR-22 is related to lupus nephritis with low eGFR. Zhang et al. [119] confirmed the positive correlation of plasma miR-200c-5p with SLEDAI scores and proteinuria, and that of miR-200b-3p with proteinuria, and negative correlation of miR-141 with serum creatinine and SLEDAI scores. Nakhjavani et al. [120] demonstrated that plasma levels of miR-21, miR-150, and miR-423 are associated with lupus nephritis with renal fibrosis.

The role of cell-free lncRNAs as biomarkers for disease or disease activity has also been explored. Abd-Elmawla et al. [121] identified the roles of plasma cell-free lncRNA, ANRIL (antisense non-coding RNA in the INK4 locus), NOS3-AS, and APOA1-AS expression in the development of vasculopathy and inflammation in SLE patients. They found increased plasma levels of ANRIL were positively associated with menopause and SLEDAI and negatively correlated with C3 levels. NOS3-AS had a negative correlation with NOX and HDL but a positive correlation with LDL-C, hypertension, and metabolic syndrome. APOA1-AS had a negative correlation with HDL-C and a positive correlation with LDL-C as well as metabolic syndrome, adhesion molecule expression, and oxidized low-density lipoprotein, OXLDL. They have concluded that the three lncRNAs play a pivotal role in the development of atherosclerosis via their atherogenic and inflammatory effects. Besides, Wu et al. [122] confirmed that decreased lncRNAs, GAS5, lnc7074 and increased lnc0597, lnc0640, and lnc5150 in plasma could be used as SLE biomarkers. The co-expression analysis showed that GAS5, lnc0640, and lnc5150 might participate in the lupus pathogenesis via the signaling pathway. Despite the above-mentioned observation, Xu et al. [123] provided a novel mitogen activated protein kinase (MAPK) signaling pathway study of lncRNA-related and miR-related competing endogenous RNAs (ceRNAs) networks for the exploration of lupus pathogenesis. They deduced that the differentially expressed lncRNAs (DE lncRNAs), myocardial infarction associated transcript (MIAT) and nuclear enriched abundant transcript -1 (NEAT1), and three novel miRs (hsa-miR-145, hsa-miR-17, and hsa-miR-143) play crucial roles in lupus pathogenesis. It is worthy to note that some of the same cell-free lncRNAs may be implicated in different autoimmune or rheumatic inflammatory diseases, among which a decreased expression of lncRNA growth arrest-specific 5 (GAS5) has been found in SLE, pediatric inflammatory bowel disease [124,125], and rheumatoid arthritis [126]. Lucafò et al. [125] unveiled that down-regulation of GAS5 can up-regulate both MMP2 and MMP9 production to induce tissue inflammation and destruction. Another lncRNA, NEAT1, is up-regulated in the serum of both SLE and multiple sclerosis patients [127]. Ma et al. [128] have reported that down-regulation of lncRNA NEAT1 can inhibit mouse renal mesangial cell proliferation, tissue fibrosis, and inflammation, whereas cell apoptosis is, on the contrary, promoted in diabetic nephropathy. Recently, Dong et al. [129] have demonstrated that lncRNA NEAT1 is overexpressed in the granulocytic myeloid-derived suppressor cells in autoimmune MRL/*lpr* mice. These cells can induce IFN-1 signaling activation of B cell via B cell-activating factor, BAFF, to promote autoantibodies production. These findings may support the hypothesis that a common etiologic factor exists in different autoimmune and inflammatory diseases.

**Figure 2:**
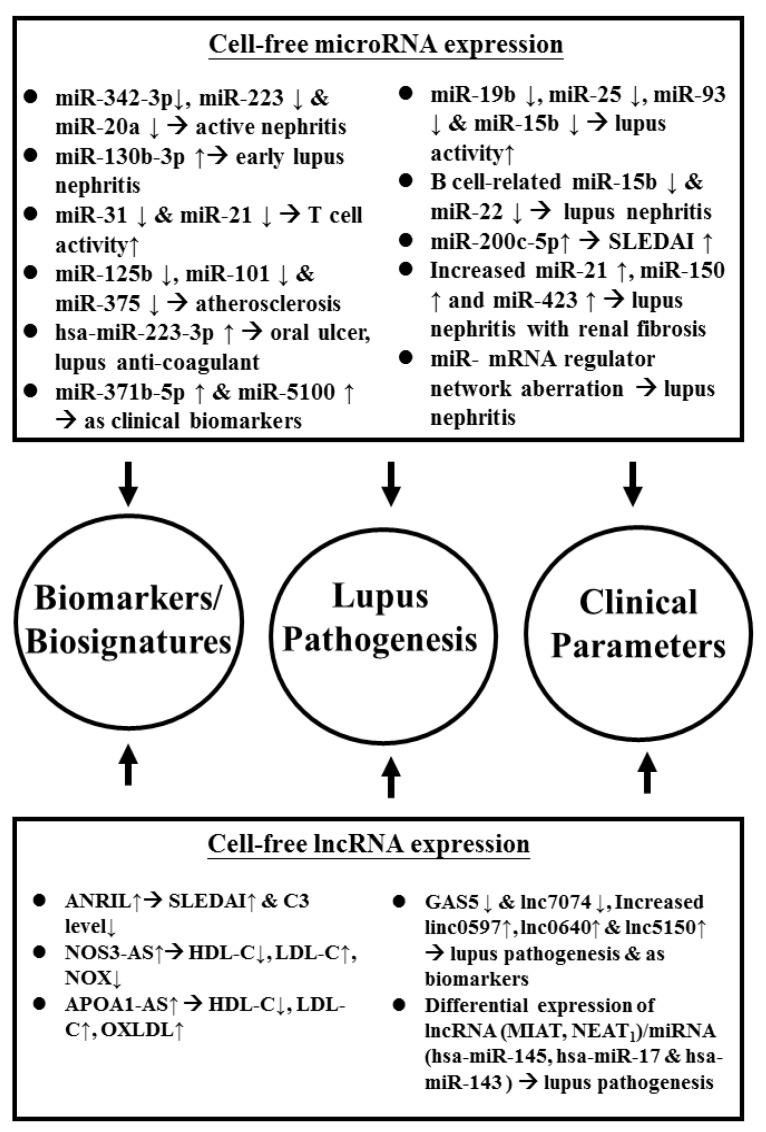
Aberrant cell-free ncRNA expression implicated in the pathogenesis, disease biomarkers/biosignatures, and different clinical parameters in patients with SLE. APOA1: apoprotein A1; AS: atherosclerosis; hsa: *Homo sapiens*; ANRIL: antisense non-coding RNA in the INK4 locus; MIAT: myocardial infarction associated transcript; NEAT: nuclear enriched abundant transcript; OS3: nitric oxide synthase 3; NOX: nitric oxide. See the other abbreviations in the main text.

Figure 2 summarizes the roles of the aberrant cell-free miR and lncRNA expressions in the lupus pathogenesis and as biomarkers/biosignatures of SLE disease activity and lupus nephritis.

Although some EV-associated miRs have been regarded as biomarkers for kidney damage in SLE, some authors in recent years have confirmed that urinary exosomes are the stable source of miR biomarkers in lupus nephritis.

#### 2.3.4. Imbalanced Urinary Cell-Free ncRNA Expression in SLE Patients with Nephritis

**Figure 3:**
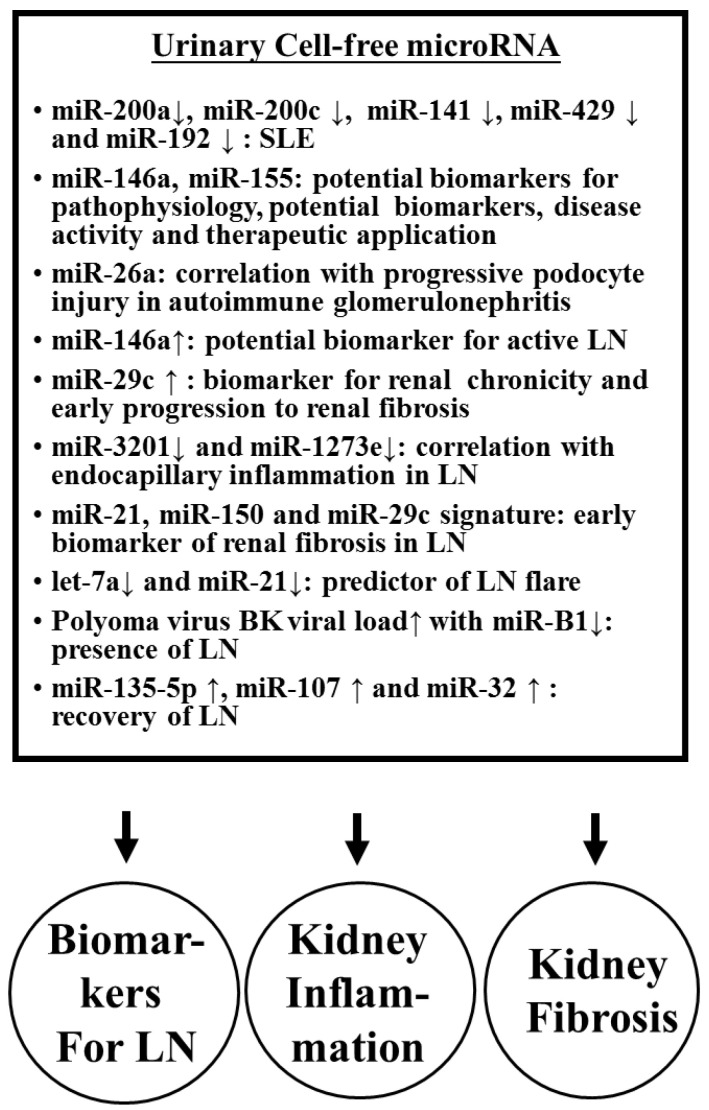
The effect of urinary cell-free miRs on the inflammation and fibrosis in lupus nephritis (LN) and as biomarkers/biosignatures for SLE and LN.

A number of recent studies revealed that imbalanced expression of many miR’s is present in the PBMCs and kidney tissue of SLE patients. Therefore, the eligibility for the detection of miR expression in urine as a liquid biopsy and useful biomarkers for lupus nephritis (LN) was fervently debated. Wang et al. [130] demonstrated decreased urinary cell-free miR-200a, miR-200c, miR-141, miR-409, and miR-192 in SLE patients. Later, the same group reported that urinary miR-146a and miR-155 would play important roles in lupus pathophysiology and could become potential biomarkers for diagnosis, a disease activity monitoring tool, and therapeutic agents or targets in SLE [131]. In regards to correlating urinary miR expression and kidney inflammation, Ichii et al. [132] explored epigenetics and found that decreased miR-26a expression correlated with the progression of podocyte injury in autoimmune glomerulonephritis. Perez-Hernandez et al. [133] detected urinary cell-free miRs and concluded that increased miR-146a discriminates out the presence of active LN and can be a potential non-invasive disease biomarker. Sole et al. [134] found miR-29c in urine correlates with the degree of nephritis chronicity and could be used as a novel non-invasive biomarker to indicate the early progression of LN to renal fibrosis in SLE patients. Cardenas-Gondalez et al. [135] profiled 2401 urinary cell-free miRs and demonstrated that down-regulation of miR-3201 and miR-1273e correlated with endocapillary inflammation in LN. Sole et al. [136] studied the biopsy-proven LN patients and concluded that cell-free urinary miR (miR-21, miR-150, and miR-29c) signatures could be used for early diagnosis of renal fibrosis in LN. Tangtanatakul et al. [137] further confirmed that down-regulation of let-7a and miR-21 in urine exosomes obtained from LN could predict disease flare. Interestingly, Li et al. [138] studied the relationships between polyomavirus BK infection (BKV) and lupus pathogenesis. They have concluded that an elevated urinary BKV viral load with a decreased miR-B1 level implies the presence of LN. From a therapeutic point of view, hypoxia-inducible factor (HIF)-1α is regarded as a potential common target for LN treatment. HIF-1α inhibitor, miR-206, can reduce nonspecific mesangial proliferation as well as IL-8, C-C chemokine ligand (CCL)2, CCL3, and C-X-C chemokine ligand (CXCL)1-induced mesangial cell proliferation, and IL-6/vascular cell adhesion molecule (VCAM)-1 expression in endothelial cells [139]. Garcia-Vives et al. [140] proved that urinary exosomal miR-135g-5p, miR-107, and miR-31 are effective novel markers for HIF-1α inhibition to predict clinical outcomes and LN recovery. Figure 3 summarizes the urinary cell-free miR expression in the pathogenesis, or as biomarkers/biosignatures and kidney fibrosis in patients with LN.

A critical review of aberrant expression of intracellular, circulating, and urinary exosomal miRs to target mRNAs and their pathological effects in patients with SLE have been made [62].

#### 2.3.5. Aberrant ncRNA Expression in the Kidney Tissues of Patients with LN

It is conceivable that differential expression of miRNAs in peripheral blood, urine, and kidney tissues occurs in patients with LN. Lu et al. [141] studied the intra-renal expression of miRNAs that included glomerular and tubular interstitial tissues in LN. They found lower glomerular and higher tubulointerstitial (TI) expression of miR-638. Both glomerular and TI expression of miR-198 was higher than in normal kidney, whereas higher miR-146a was only found in lupus glomeruli. For clinical correlation, the group noted that higher miR-638 expression in TI was significantly correlated with proteinuria and disease activity, while higher glomerular miR-146a expression was correlated with estimated GFR and histological activity index. In addition to kidney inflammation, some miRs also show profibrotic or anti-fibrotic effects. Zhou et al. [142] have compared miR expression in biopsied renal tissues from LN and found that miR-150 positively correlated to chronicity scores and profibrotic protein expression in both proximal tubules and mesangial cells through downregulation of *SOCS1*. Furthermore, Krasoudaki et al. [143] have demonstrated 24 miRs were dysregulated in human LN tissues, among which miR-422a showed the highest upregulation (17 fold) in active LN with fibrinoid necrosis. Their transfection studies showed that miR-422a directly targets kallikrein-related peptidylase 4 (*KLK4*) mRNA, a serine esterase with putative renal protective activity. On the contrary, Costa-Reis et al. [144] showed that miR-76a and miR-30b were decreased in kidney and urine of LN patients. These two miRs may control IFN-α induced human epidermal growth factor receptor 2 (HER-2) mediated mesangial cell proliferation. The lack of these two miRs may lead to proliferative LN. In the same regard, Yao et al. [145] demonstrated that down-regulated hsa-miR-371-5p in human mesangial cells from LN might promote proliferation and decrease the apoptosis of glomerular mesangial cells. The group also identified HIF-1α as the direct target gene of hsa-miR-371-5p in mesangial cells. The overexpression of this miR would ameliorate mesangial cell proliferation in LN. In an animal experiment, Liu et al. [146] found that the expression of miR-410 in kidney tissue of MRL/*lpr* mice was decreased compared to that in BALB/C mice, whereas IL-6 was overexpressed in the same model of MRL/*lpr*. Luciferase assay also showed miR-410 could directly bind to the 3′-UTR region of IL-6 gene to suppress IL-6 production. The over-expression of miR-410 significantly suppressed TGF-β1 and collagen I/III gene expression in a cell model. These results have suggested that miR-410 can be used to indicate suppressed renal fibrosis in LN. Another animal study led by Leiss et al. [147], by using pristane-induced miR-155 deficient mice, demonstrated that miR-155 deficient mice had significantly less pulmonary and renal diseases associated with lower serum autoantibodies and decreased T_h2_, T_h17,_ and CD4^+^CD25^+^(FoxP3^−^) cells. A similar study conducted by Kong et al. [148] showed that transfection of the miR-155 into the cultured cells might create a condition mimicking CXCL-13-treated human glomerular mesangial cells and result in a significantly reduced proliferation rate due to decreased phosphorylation of the CXCR5-extracellular signal- regulated kinase (ERK) signaling pathway as well as TGF-β1 production. Li et al. [149] injected miR-183 into MRL/*lpr* mice and found that the procedure could result in a reduction of anti-DNA autoantibodies and immune complexes in the autoimmune lupus mice associated with restoration of their Treg and Th17 cell populations and prolonged their survival via targeting of the mammalian target of rapamycin (mTOR) pathway.

**Figure 4:**
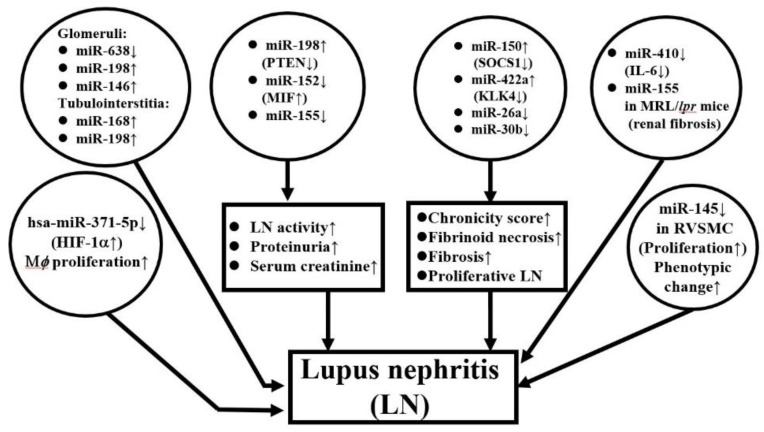
ncRNA expression in lupus nephritis tissue. hsa: *Homo sapiens*; PTEN: Phosphatase and tensin homolog deleted on chromosome ten; MIF: Macrophage migration inhibitory factor; SOCS: Suppressor of cell signaling; HIF: Hypoxia-inducible factor; M*ϕ*: macrophage; RVSMC: rat vascular smooth muscle cell.

In contrast, Cui et al. [150] observed that higher expression of miR-198 in lupus renal tissue was correlated with disease activity. In addition, miR-198 could directly bind to phosphatase and a tensin homolog deleted on chromosome ten (PTEN) 3′-untranslated region. Thus, miR-198 may promote the proliferation of glomerular mesangial cells to contribute to SLE progression by targeting PTEN. Zheng et al. [151] reported that down-regulated miR-152 expression in LN tissue was inversely correlated to 24 h urine protein excretion and serum creatinine and could directly target macrophage migration inhibitory factor (MIF). Transfection of miR-152 may alleviate LN severity and chronicity index through down-regulation of MIF-induced *COL1A1*. By studying juvenile LN, Cai et al. [152] have demonstrated that decreased expression of miR-145 in renal vascular smooth muscle cells increased the vascular damage in LN. A functional study revealed miR-145 can suppress *PDGF-BB* induced cell proliferation, migration, and phenotypic differentiation of human vascular smooth muscle cells and may become a new target for treating renal vascular lesions in LN. Figure 4 depicts the crucial ncRNAs expression in kidney tissues to reflect their pathogenetic roles in patients with LN.

#### 2.3.6. Circular RNAs Expression Profile in SLE

Circular RNAs (circRNAs) are a group of ncRNAs forming covalently closed RNA circles that are derived from exon, intron, untranslated or intergenic regions of the genome in mammals [153]. circRNAs can regulate gene expression by acting as competitive endogenous RNAs (ceRNAs) to serve as miRNA sponges for the sequestration of miRNAs from modulating gene transcription. Although accumulating data have supported the involvement of circRNAs in a number of human cancers such as hepatocellular, esophageal, and gastrointestinal carcinomas [154,155,156,157], only a few reports have identified abnormal circRNA expression in patients with SLE.

**Figure 5:**
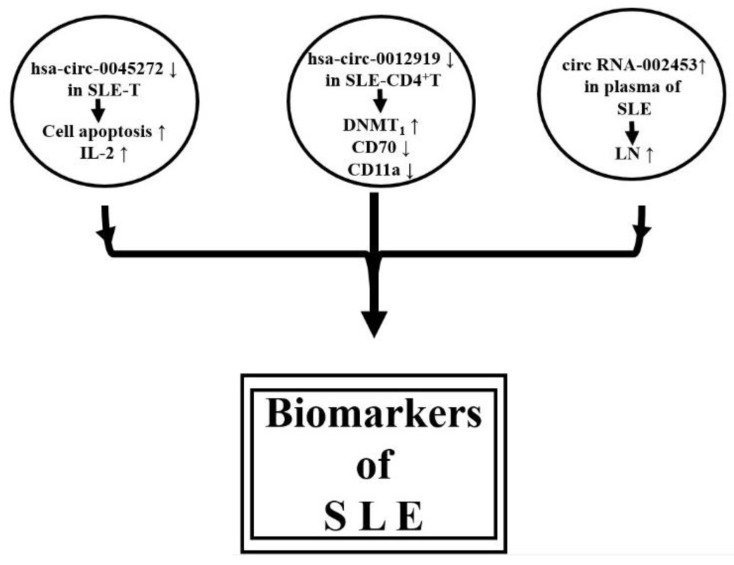
Proposed acting mechanisms of circRNAs in the pathogenesis and as biomarkers in SLE.

Li et al. [158] were the first group to speculate the roles of circRNAs in wiping up miRs in the initiation and progression of SLE. Later, the group revealed that down-regulation of hsa-circ-0045272 in SLE-T cells potentially enhances cell apoptosis and IL-2 production [159]. Zhang et al. [160] have isolated CD4^+^ T cells from SLE patients and conducted circRNA microarray analysis. They identified that down-regulated hsa-circ-0012919 increased DNMT1 expression, reduced CD70 and CD11a expression, and reversed the DNA hypomethylation of CD11a and CD70 in SLE-CD4+T cells. The molecular mechanism of hsa-circ-0012919 relied on the regulation of *KLF13* and *RANTES* by sponging (absorbing) miR-125a. It was suggested that hsa-circ-0012919 could be used as a biomarker for SLE. Furthermore, Quyang et al. [161] analyzed the plasma from patients with LN and demonstrated that upregulation of plasma circRN002453 in LN patients was related to nephritis severity and could become a potential biomarker for LN diagnosis. Li et al. [162] have even suggested that comprehensive circRNA profiling of plasma could be used as novel biosignatures for SLE. Cortes et al. [163] concluded that circRNAs are not only involved in the physiology and pathophysiology of acute and chronic inflammation but can serve as novel biomarkers/biosignatures for the disease activity of SLE. Figure 5 summarizes the possible acting mechanisms of circRNAs in pathogenesis and as biomarkers in SLE.

## 3. Conclusions

Recent investigations have revealed that intracellular and extracellular ncRNAs, including miRs, lncRNAs, and circRNAs, are the key molecules for post-transcriptional regulation of mRNA expression. The extracellular ncRNAs include free and exosomal ncRNAs, which are distributed in plasma, urine, saliva, and other body fluids to mediate remote cell-cell or cell-tissue communication. The aberrant ncRNA expression may derange the immune-related cell functions and lead to autoimmunity. SLE, as an archetype of systemic autoimmune disease, is found to have multi-organ damages with a diversely deranged ncRNA expression associated with a wide spectrum of immune dysfunctions. However, only a bunch of ncRNA expression profiling data has been reported in the literature without consistency. Moreover, the cause-effective relationships between deranged ncRNA expression and functional abnormalities remain largely uncertain. It is expected that unified microarray analysis kits can probably solve this problem. Since lncRNAs and circRNAs may serve as sponges for miRs, the interactions between lncRNA and miR and between circRNA and miR are interesting issues for unraveling post-transcriptional regulation in the future. It is expected that more lncRNAs and circRNAs will be identified for understanding the pathophysiological roles or for taking as biomarkers/biosignatures in patients with SLE and LN. To utilize ncRNA mimics or inhibitors as tools for targeting the lesional focus will also become a novel therapeutic strategy in the treatment of SLE.

## 4. Prospective

LN is one of the severe complications in patients with SLE. It is expected that urinary exosomal and cell-free ncRNAs may directly reflect the pathophysiology and represent as pathological biomarkers in LN. In conjunction with ncRNA expression in LN tissues in situ, the urinary stable exosomal ncRNA expression would become more specific and reliable biomarkers for the diagnosis, monitoring, and finally therapeutic effectiveness in patients with LN.

The intrinsic and environmental factors such as mental stresses, hormones, infections, and other random speculative factors in the initiation of ncRNA dysregulation in SLE need further investigation.

## Figures and Tables

**Table 1:** Abnormal cell biology and aberrant intracellular signaling of immune-related cells in lupus pathogenesis.

Parameter	Immunological Function	References
**[I] Innate immune cells:**		
Macrophage/Dendritic cells	Phagocytosis↓	[1,2,3,17]
	Lymphocyte stimulation↑	
	Pro-inflammatory cytokine production↑:	
	IL-1β, IL-6, TNF-α, IFN-α	
Natural killer cells	Cytotoxicity↓	[5,6,18,19]
	Proliferation↓	
	Immunosuppression↓	
Neutrophils	Phagocytosis↓	[4,16]
	NET formation↑	
	Effects on Th1 cytokine production↓	
	Effects on Th2 cytokine production↑	
**[II] Adaptive immune cells:**		
T lymphocytes	Th1/Th2 ratio↓	[11,12,20,21,22,23,24]
	Th17/Treg ratio↑	
	T follicular helper cell number↑	
	CD45 RO^+^ memory T cell number↑	
B lymphocytes	Autoantibody production↑	[13,14,15,25,26,27]
	CD5^+^ B cell number↑	
	Breg number↓	
**[III] Intracellular signaling:**		
SOCS system STAT signaling IFN-1 signaling Ubiquitin regulation for IFN-γ	SOCS1 mRNA and signaling↓ (?) ↑ (?)	[29,30] [31] [32,33] [34]
**[IV] Immunometabolism**	Bioenergetics↓	[35]
**[V] Redox capacity** ● Oxidative stresses	↓ ↑	[36,37,38,39,40]
● Mitochondrial DNA heteroplasmy	↑	

↑: increase;↓: decrease; Treg: regulatory T cell; Breg: regulatory B cell; CD: cluster of differentiation; STAT: signal transducer and activator of transcription; IFN: interferon; IFN-1: type 1 interferon; NET: neutrophil extracellular trap; SOCS: suppressor of cytokine signaling.

**Table 2:** Genetic loci associated with lupus pathogenesis demonstrated in the big data from genome-wide association studies.

**[I] Advances in genome-wide association studies on SLE [43,44,45,46,47,48,49,50,51,52,53].**		
● Immune complex processing genes:		
•	*C1q, C2, C4*	
•	*FcγR2A, FcγR3A*	
•	*CRP*	
•	*ITGAM*	
● Immune signal transduction genes:		
•	*STAT4*	• *TNFSF4*
•	*IRF5*	• *BLK*
•	*BAN K1*	• *MECP2 (?)*
•	*PTPN22*	• *PXK (?)*
•	*PCDCD1*	• *XKR6 (?)*
● TLR/IFN-1 pathway:		
•	*TREX1*	• *IRAK1 (?)*
•	*TNFAIP3*	• *STAT1 (?)*
● HLA-DR:		
•	Disease-association: DR3, DR9, DR15, DQA1*0101	
•	Disease-protection: *DR4, DR11, DR14*	
● Others:		
•	*PDHX/CD44*	• *IFNK*
•	*ICAM1-ICAM4-ICAM5*	• *UBE2 L3*
•	*TRAF6*	• *IRF8*
•	*PPP2CA*	• *MAV5*
•	*MYG9-APOL1*	
● IFN-1 signaling:		
•	*IRF4*	• *MYC*
•	*IRF5*	• *IFIH1* (MDA5)
•	*ERK1*	• *TNFAIP3*
● B cell receptor signaling:		
•	*FcγRIIb* (FcγRIIB)	• *PRDM1* (BLIMP1)
•	*BANK1* (BANK1)	• *CSK* (C-Src tyrosine kinase, CSK)
•	*BLK* (BLK)	

The molecules in ( ) are encoded by the respective genes. SLE: systemic lupus erythematosus; IFN-1: type 1 interferon; TLR: Toll-like receptor; HLA: human leukocyte antigen.

**Table 3:** Epigenetic regulations of genes in patients with SLE.

**[I] DNA hypomethylation in CD4+ T cells [63,64,65,66,67,68,69,70]**		
	● *ITGAL* (CD11a)	● *PT PRC* (CD45)
	● *PRF1* (perforin)	● *UHRF1 BP1*
	● *TNF SF7* (CD70)	● *IRF5*
	● *TNF SF5* (CD40L)	● *IKZF3*
	● *PP2A* cα	● *UBE2L3*
	● *IRF7* (IFN-1 master regulatory gene)	● MHC-class III ● HLA-*DQβ2*
**[II] DNA hypomethylation in lupus nephritis specimen [64,71]**		
	● *IFNγR*	● *IFI35*
	● *STAT1*	● *IRF7*
**[III] Increased DNA hydroxymethylation in CD4+ T cells [72,73,74]**		
	● *TREX1*	● *SOCS1*
	● *CDKN1A*	● *NR2F*
	● *CDKN1B*	● *IL15RA*
		● *CTCF*

The molecules in ( ) denote the proteins encoded by the respective genes; CD: cluster of differentiation; HLA: human leukocyte antigen; MHC: major histocompatibility complex; IFN-1: type 1 interferon.

**Table 4:** Histone and non-histone protein modifications in immune-related cells and intracellular molecules in patients with SLE.

**[I]**	**Histone modifications in SLE**
	● Global histone H3 and H4 hyperacetylation in active lupus CD4^+^ T cells [75,76]
	● H3K4me variants in PBMC of SLE [76]
	● Increased H4 acetylation in SLE-monocytes [77]
	● Elevated histone H3 acetylation and dimethylated H3 lysine 4 (H3K4me2) levels in lupus CD4^+^ T cells [78]
	● Increased H3K18ac and reduced H3K27me3 of IL-17 gene cluster in SLE-T cells [79]
	● H3K18 deacetylation and H3K27 trimethylation of IL-2 in CD3^+^ T, CD4^+^ T and effector (CD4^+^) T cells in SLE [79,80,81]
	● H3K18ac elevation for IL-10 in CD3^+^ and CD4^+^ T cells in SLE [79,80,81]
	● H3ac elevation for TNF-α in SLE-monocytes [79,80,81]
	● H3K4me elevation for IRF1 in SLE-monocytes [82]
**[II]**	**Non-histone protein modifications in patients with SLE**
	● U1 small nuclear ribonucleoprotein 68 k [83]
	● MAPK signaling molecules [84]
	● Complex ribonuclear proteins SSA/Ro and SSB/La [84,85]
	● Spliceosomal Sm protein [86,87]
	● Serine/arginine rich splicing factor 1 (SRSF1) [88]
	● Neutrophilic myeloperoxidase

H: histidine; K: lysine; R: arginine; ac: acetylation; me: methylation; CD: cluster of differentiation; PBMC: peripheral blood mononuclear cell; me: methylation; ac acetylation; MAPK: mitogen activated protein kinase; Sm: Smith.

## References

[1] Rönnblom L., Pascual V. (2008). The innate immune system in SLE: Type I interferons and dendritic cells. Lupus.

[2] Katsiari C.G., Liossis S.-N.C., Sfikakis P.P. (2010). The pathophysiologic role of monocytes and macrophages in systemic lupus erythematosus: A reappraisal. Semin. Arthritis Rheum..

[3] Kim J.-M., Park S.-H., Kim H.-Y., Kwok S.-K. (2015). A plasmacytoid dendritic cells-type I interferon axis is critically implicated in the pathogenesis of systemic lupus erythematosus. Int. J. Mol. Sci..

[4] Tsai C.-Y., Li K.-J., Hsieh S.-C., Liao H.-T., Yu C.-L. (2019). What’s wrong with neutrophils in lupus?. Clin. Exp. Rheumatol..

[5] Spada R., Rojas J.M., Barber D.F. (2015). Recent findings on the role of natural killer cells in the pathogenesis of systemic lupus erythematosus. J. Leukoc. Biol..

[6] Cruz-González D.d.J., Gómez-Martin D., Layseca-Espinosa E., Baranda L., Abud-Mendoza C., Alcocer-Varela J., González-Amaro R., Monsiváis-Urenda A.E. (2018). Analysis of the regulatory function of natural killer cells from patients with systemic lupus erythematosus. Clin. Exp. Immunol..

[7] Weidenbusch M., Kulkarni O.P., Anders H.-J. (2017). The innate immune system in human systemic lupus erythematosus. Clin. Sci..

[8] Leffler J., Bengtsson A.A., Blom A.M. (2014). The complement system in systemic lupus erythematosus: An update. Ann. Rheum. Dis..

[9] Liu T., Son M., Diamond B. (2020). HMGB1 in systemic lupus erythematosus. Front. Immunol..

[10] Liu Y., Lightfoot Y.L., Seto N., Carmona-Rivera C., Moore E., Goel R., O’Neil L., Mistry P., Hoffmann V., Mondal S. (2018). Peptidylarginine deiminases 2 and 4 modulate innate and adaptive immune responses in TLR-7-dependent lupus. JCI Insight.

[11] Moulton V.R., Tsokos G.C. (2015). T cell signaling abnormalities contribute to aberrant immune cell function and autoimmunity. J. Clin. Investig..

[12] Katsuyama T., Tsokos G.C., Moulton V.R. (2018). Aberrant T cell signaling and subsets in systemic lupus erythematosus. Front. Immunol..

[13] Morawski P.A., Bolland S. (2017). Expanding the B cell centric view of systemic lupus erythematosus. Trends Immunol..

[14] Yap D.Y.H., Chan T.M. (2019). B cell abnormalities in systemic lupus erythematosus and lupus nephritis—Role in pathogenesis and effect of immunosuppressive treatments. Int. J. Mol. Sci..

[15] Wang T., Marken J., Chen J., Tran V.B., Li Q.-Z., Li M., Cerosaletti K., Elkon K.B., Zeng X., Giltiay N.V. (2019). High *TCR*7 expression drives the expansion of CD19^+^CD24^hi^CD38^hi^ transitional B cells and autoantibody production in SLE patients. Front. Immunol..

[16] O’Neil L.J., Kaplan M.J., Carmona-Rivera C. (2019). The role of neutrophils and neutrophil extracellular traps in vascular damage in systemic lupus erythematosus. J. Clin. Med..

[17] Mackern-Oberti J.P., Llanos C., Riedel C.A., Bueno S.M., Kalergis A.M. (2015). Contribution of dendritic cells to the autoimmune pathology of systemic lupus erythematosus. Immunology.

[18] Hervier B., Beziat V., Haroche J., Mathian A., Lebon P., Ghillani-Dalbin P., Musset L., Debré P., Amoura Z., Vieillard V. (2011). Phenotype and function of natural killer cells in systemic lupus erythematosus: Excess interferon-γ production in patients with active disease. Arthritis Rheum..

[19] Henriques A., Teixeira L., Inês L., Carvalheiro T., Gonçalves A., Martinho A., Pais M.L., da Silva J.A.P., Paiva A. (2013). NK cells dysfunction in systemic lupus erythematosus: Relation to disease activity. Clin. Rheumatol..

[20] Rose T., Dörner T. (2017). Drivers of the immunopathogenesis in systemic lupus erythematosus. Best Pract. Res. Clin. Rheumatol..

[21] Kubo S., Nakayamada S., Yoshikawa M., Miyazaki Y., Sakata K., Nakano K., Hanami K., Iwata S., Miyagawa I., Saito K. (2017). Peripheral immunophenotyping identifies three subgroups based on T cell heterogeneity in lupus patients. Arthritis Rheumatol..

[22] Álvarez-Rodríguez L., Martínez-Taboada V., Calvo-Alén J., Beares I., Villa I., López-Hoyos M. (2019). Altered Th_17_/Treg ratio in peripheral blood of systemic lupus erythematosus but not primary antiphospholipid syndrome. Front. Immunol..

[23] Yang S., Hu C., Huang B., Zhang L., Li Q., Jiang S., Zhang Q., Liu J., Zhang X., Tan J. (2004). Aberration of CCR_7_CD8 memory T cells from patients with systemic lupus erythematosus: An inducer of T helper type 2 bias of CD4 T cells. Immunology.

[24] Blanco P., Ueno H., Schmitt N. (2016). T follicular helper (Tfh) cells in lupus: Activation and involvement in SLE pathogenesis. Eur. J. Immunol..

[25] Becker H., Weber C., Storch S., Federlin K. (1990). Relationship between CD5^+^ B lymphocytes and the activity of systemic autoimmunity. Clin. Immunol. Immnopathol..

[26] Omar H.H., Nasef S.I., Omar H.H., Ghaly M.S. (2017). CD5+ B lymphocytes in systemic lupus erythematosus patients: Relation to disease activity. Clin. Rheumatol..

[27] Wang T., Mei Y., Li Z. (2019). Research progress on regulatory B cells in systemic lupus erythematosus. BioMed Res. Int..

[28] Pan L., Lu M.-P., Wang J.-H., Xu M., Yang S.-R. (2020). Immunological pathogenesis and treatment of systemic lupus erythematosus. World J. Pediatr..

[29] Qiu L.-J., Xu K., Liang Y., Cen H., Zhang M., Wen P.-F., Ni J., Xu W.-D., Leng R.-X., Pan H.-F. (2015). Decreased SOCS1 mRNA expression levels in peripheral blood mononuclear cells from patients with systemic lupus erythematosus in a Chinese population. Clin. Exp. Med..

[30] Wang H., Wang J., Xia Y. (2017). Defective suppressor of cytokine signaling 1 signaling contributes to the pathogenesis of systemic lupus erythematosus. Front. Immunol..

[31] Goropevšek A., Holcar M., Avčin T. (2017). The role of STAT signaling pathways in the pathogenesis of systemic lupus erythematosus. Clin. Rev. Allergy Immunol..

[32] Ramírez-Vélez G., Medina F., Ramírez-Montaño L., Zarazúa-Lozada A., Hernández R., Llorente L., Moreno J. (2012). Constitutive phosphorylation of interferon receptor A-associated signaling proteins in systemic lupus erythematosus. PLoS ONE.

[33] Wu L., Qin Y., Xia S., Dai M., Han X., Wu Y., Zhang X., Ma J., Wang Y., Tang Y. (2016). Identification of cyclin-dependent kinase 1 as a novel regulator of type I interferon signaling in systemic lupus erythematosus. Arthritis Rheumatol..

[34] Yu Y., Su Z., Wang Z., Xu H. (2017). USP_7_ is associated with greater disease activity in systemic lupus erythematosus via stabilization of the IFNα receptor. Mol. Med. Rep..

[35] Li K.-J., Wu C.-H., Hsieh S.-C., Lu M.-C., Tsai C.-Y., Yu C.-L. (2012). Deranged bioenergetics and defective redox capacity in T lymphocytes and neutrophils are related to cellular dysfunction and increased oxidative stress in patients with systemic lupus erythematosus. Clin. Dev. Immunol..

[36] Lee H.-T., Lin C.-S., Chen W.-S., Liao H.-T., Tsai C.-Y., Wei Y.-H. (2012). Leukocyte mitochondrial DNA alteration in systemic lupus erythematosus and its relevance to the susceptibility to lupus nephritis. Int. J. Mol. Sci..

[37] Lee H.-T., Wu T.-H., Lin C.-S., Lee C.-S., Wei Y.-H., Tsai C.-Y., Chang D.-M. (2016). The pathogenesis of systemic lupus erythematosus-from the viewpoint of oxidative stress and mitochondrial dysfunction. Mitochondrion.

[38] Lee H.-T., Wu T.-H., Lin C.-S., Lee C.-S., Pan S.-C., Chang D.-M., Wei Y.-H., Tsai C.-Y. (2017). Oxidative DNA and mitochondrial DNA change in patients with SLE. Front. Biosci..

[39] Lee H.-T., Lin C.-S., Pan S.-C., Wu T.-H., Lee C.-S., Chang D.-M., Tsai C.-Y., Wei Y.-H. (2019). Alterations of oxygen consumption and extracellular acidification rates by glutamine in PBMCs of SLE patients. Mitochondrion.

[40] Tsai C.-Y., Shen C.-Y., Liao H.-T., Li K.-J., Lee H.-T., Lu C.-S., Wu C.-H., Kuo Y.-M., Hsieh S.-C., Yu C.-L. (2019). Molecular and cellular bases of immunosenescence, inflammation, and cardiovascular complications mimicking “inflammaging” in patients with systemic lupus erythematosus. Int. J. Mol. Sci..

[41] Almlöf J.C., Alexsson A., Imgenberg-Kreuz J., Sylwan L., Bäcklin C., Leonard D., Nordmark G., Tandre K., Eloranta M.L., Padyukov L. (2017). Novel risk genes for systemic lupus erythematosus predicted by random forest classification. Sci. Rep..

[42] Julià A., López-Longo F.J., Venegas J.J.P., Bonàs-Guarch S., Olivé A., Andreu J.L., Aquirre-Zamorano M.Á., Vela P., Nolla J.M., da la Fuente J.L.M. (2018). Genome-wide association study meta-analysis identifies five new loci for systemic lupus erythematosus. Arthritis Res. Ther..

[43] Regunathan-Shenk R., Radhakrishnan J. (2018). Pathogenesis of SLE nephritis in the era of precision medicine. Curr. Rheumatol. Rev..

[44] Goulielmos G.N., Zervou M.I., Vazgiourakis V.M., Ghodke-Puranik Y., Garyfallos A., Niewold T.B. (2018). The genetics and molecular pathogenesis of systemic lupus erythematosus (SLE) in populations of different ancestry. Gene.

[45] Kwon Y.-C., Chun S., Kim K., Mak A. (2019). Update on the genetics of systemic lupus erythematosus: Genome-wide association studies and beyond. Cells.

[46] Fike A.J., Elcheva I., Rahman Z.S.M. (2019). The post-GWAS era: How to validate the contribution of gene variants in lupus. Curr. Rheumatol. Rep..

[47] Deng Y., Tsao B.P. (2014). Advances in lupus genetics and epigenetics. Curr. Opin. Rheumatol..

[48] Rawlings D.J., Metzler G., Wray-Dutra M., Jackson S.W. (2017). Altered B cell signalling in autoimmunity. Nat. Rev. Immunol..

[49] Gorradin O., Cohen A.J., Luppino J.M., Bayles I.M., Schumacher F.R., Scacheri P.C. (2016). Modeling disease risk through analysis of physical interactions between genetic variants within chromatin regulatory circuitry. Nat. Genet..

[50] Jackson S.W., Kolhatkar N.S., Rawlings D.J. (2015). B cells take the front seat: Dysregulated B cell signals orchestrate loss of tolerance and autoantibody production. Curr. Opin. Immunol..

[51] Bronson P.G., Chaivorapol C., Ortmann W., Behrens T.W., Graham R.R. (2012). The genetics of type I interferon in systemic lupus erythematosus. Curr. Opin. Immunol..

[52] Catalina M.D., Owen K.A., Labonte A.C., Grammer A.C., Lipsky P.E. (2020). The pathogenesis of systemic lupus erythematosus: Harnessing big data to understand the molecular basis of lupus. J. Autoimmun..

[53] Lever E., Alves M.R., Isenberg D.A. (2020). Towards precision medicine in systemic lupus erythematosus. Pharm. Pers. Med..

[54] Fielder A.H.L., Walport M.J., Batchelor J.R., Rynes R.I., Black C.M., Dodi I.A., Hughes G.R.V. (1983). Family study of the major histocompatibility complex in patients with systemic lupus erythematosus: Importance of null alleles of C4A and C4B in determining disease susceptibility. Br. Med. J. (Clin. Res. Ed.).

[55] Liu Z., Davidson A. (2012). Taming lupus—A new understanding of pathogenesis is leading to clinical advances. Nat. Med..

[56] Alarcón G.S., McGwin G., Petri M., Ramsey-Goldman R., Fessler B.J., Vilá L.M., Edberg J.C., Reveille J.D., Kimberly R.P., PROFILE study group (2006). Time to renal disease and end-stage renal disease in PROFILE: A multiethnic lupus cohort. PLoS. Med..

[57] Brown E.E., Edberg J.C., Kimberly R.P. (2007). Fc receptor genes and the systemic lupus erythematosus diathesis. Autoimmunity.

[58] Morris D.L., Roberts A.L., Witherden A.S., Tarzi R., Barros P., Whittaker J.C., Cook T.H., Aitman T.J., Vyse T.J. (2010). Evidence for both copy number and allelic (NA_1_/NA_2_) risk at the FCGR_3_B locus in systemic lupus erythematosus. Eur. J. Hum. Genet..

[59] Chen J.-Y., Wang C.-M., Chang S.-W., Cheng C.-H., Wu-Jan Y.-J., Lin J.-C., Yang B., Ho H.-H., Wu J. (2014). Association of *FCGR3A* and *FCGR3B* copy number variations with systemic lupus erythematosus and rheumatoid arthritis in Taiwanese patients. Arthritis Rheumatol..

[60] Fernando M.M.A., Stevens C.R., Walsh E.C., De Jager P.L., Goyette P., Plenge R.M., Vyse T.J., Rioux J.D. (2008). Defining the role of the MHC in autoimmunity: A review and pooled analysis. PLoS Genet..

[61] Ghodke-Puranik Y., Niewold T.B. (2015). Immunogenetics of systemic lupus erythematosus: A comprehensive review. J. Autoimmun..

[62] Tsai C.-Y., Hsieh S.-C., Lu C.-S., Wu T.-H., Liao H.-T., Wu C.-H., Li K.-J., Kuo Y.-M., Lee H.-T., Shen C.-Y. (2019). Cross-talk between mitochondrial dysfunction-provoked oxidative stress and aberrant noncoding RNA expression in the pathogenesis and pathophysiology of SLE. Int. J. Mol. Sci..

[63] Zhang Y., Zhao M., Sawalha A.H., Richardson B., Lu Q. (2013). Impaired DNA methylation and its mechanisms in CD4(+) T cells of systemic lupus erythematosus. J. Autoimmun..

[64] Coit P., Renauer P., Jeffries M.A., Merrill J.T., McCune W.J., Maksimowicz-McKinnon K., Sawalha A.H. (2015). Renal involvement in lupus is characterized by unique DNA methylation changes in naïve CD4+ T cells. J. Autoimmun..

[65] Imgenberg-Kreuz J., Almlöf J.C., Leonard D., Alexsson A., Nordmark G., Eloranta M.-L., Rantapää-Dahlqvist S., Bengtsson A.A., Jönsen A., Padyukov L. (2018). DNA methylation mapping identifies gene regulatory effects in patients with systemic lupus erythematosus. Ann. Rheum. Dis..

[66] Yeung K.S., Chung B.H.-Y., Choufani S., Mok M.Y., Wong W.L., Mak C.C.Y., Yang W., Lee P.P.W., Wong W.H.S., Chen Y. (2017). Genome-wide DNA methylation analysis of Chinese patients with systemic lupus erythematosus identified hypomethylation in genes related to the type I interferon pathway. PLoS ONE.

[67] Teruel M., Sawalha A.H. (2017). Epigenetic variability in systemic lupus erythematosus: What we learned from genome-wide DNA methylation studies. Curr. Rheumatol. Rep..

[68] Weeding E., Sawalha A.H. (2018). Deoxyribonucleic acid methylation in systemic lupus erythematosus: Implications for future clinical practice. Front. Immunol..

[69] Joseph S., George N.I., Green-knox B., Treadwell E.L., Word B., Yim S., Lyn-Cook B. (2019). Epigenome-wide association study of peripheral blood mononuclear cells in systemic lupus erythematosus: Identifying DNA methylation signatures associated with interferon-related genes based on ethnicity and SLEDAI. J. Autoimmun..

[70] de la Calle-Fabregat C., Morante-Palacios O., Ballestar E. (2020). Understanding the relevance of DNA methylation changes in immune differentiation and disease. Genes.

[71] Zhang L., Zhu H., Li Y., Dai X., Zhou B., Li Q., Zuo X., Luo H. (2017). The role of IF135 in lupus nephritis and related mechanisms. Mod. Rheumatol..

[72] Ichiyama K., Chen T., Wang X., Yan X., Kim B.-S., Tanaka S., Ndiaye-Lobry D., Deng Y., Zou Y., Zheng P. (2015). The methylcytosine dioxygenase Tet2 promotes DNA demethylation and activation of cytokine gene expression in T cells. Immunity.

[73] Sui W., Tan Q., Yang M., Yang Q., Lin H., Ou M., Xue W., Chen J., Zou T., Jing H. (2015). Genome-wide analysis of 5-hmC in the peripheral blood of systemic lupus erythematosus patients using an hMeDIP-chip. Int. J. Mol. Med..

[74] Zhao M., Wang J., Liao W., Li D., Li M., Wu H., Zhang Y., Gershwin M.E., Lu Q. (2016). Increased 5-hydroxymethylcytosine in CD4(+) T cells in systemic lupus erythematosus. J. Autoimmun..

[75] Hu N., Qiu X., Luo Y., Yuan J., Li Y., Lei W., Zhang G., Zhou Y., Su Y., Lu Q. (2008). Abnormal histone modification patterns in lupus CD4^+^ T cells. J. Rheumatol..

[76] Dai Y., Zhang L., Hu C., Zhang Y. (2010). Genome-wide analysis of histone H3 lysine 4 trimethylation by ChIP-chip in peripheral blood mononuclear cells of systemic lupus erythematosus patients. Clin. Exp. Rheumatol..

[77] Zhang Z., Song L., Maurer K., Petri M.A., Sullivan K.E. (2010). Global H4 acetylation analysis by ChIP-chip in SLE monocytes. Genes Immun..

[78] Zhou Y., Qiu X., Luo Y., Yuan J., Li Y., Zhong Q., Zhao M., Lu Q. (2011). Histone modifications and methyl-CpG-binding domain protein levels at the TNFSF_7_ (CD_70_) promoter in SLE CD_4_+ T cells. Lupus.

[79] Hedrick C.M. (2017). Epigenetics in SLE. Curr. Rheumatol. Rep..

[80] Hedrich C.M. (2018). Mechanistic aspects of epigenetic dysregulation in SLE. Clin. Immunol..

[81] Farivar S., Aghamaleki F.S. (2018). Effects of major epigenetic factors on systemic lupus erythematosus. Iran. Biomed. J..

[82] Karagianni P., Tzioufas A.G. (2019). Epigenetic perspectives on systemic autoimmune disease. J. Autoimmun..

[83] Nagai K., Arito M., Takakuwa Y., Ooka S., Sato T., Kurokawa M.S., Okamoto K., Uchida T., Suematsu N., Kato T. (2012). Altered posttranslational modification on U1 small nuclear ribonucleoprotein 68k in systemic autoimmune diseases detected by 2D Western blot. Electrophoresis.

[84] Routsias J.G., Tzioufas A.G. (2010). B-cell epitopes of the intracellular autoantigens Ro/SSA and La/SSB: Tools to study the regulation of the autoimmune response. J. Autoimmun..

[85] Terzoglou A.G., Routsias J.G., Avrameas S., Moutsopoulos H.M., Tzioufas A.G. (2006). Preferential recognition of the phosphorylated major linear B-cell epitope of La/SSB 349-368 aa by anti-La/SSB autoantibodies from patients with systemic autoimmune diseases. Clin. Exp. Immunol..

[86] Brahms H., Raymackers J., Union A., de Keyser F., Meheus L., Lührmann R. (2000). The C-terminal RG dipeptide repeats of the spliceosomal Sm proteins D1 and D3 contain symmetrical dimethylarginines, which form a major B-cell epitope for anti-Sm autoantibodies. J. Biol. Chem..

[87] Zavala-Cerna M.G., Martínez-García E.A., Torres-Bugarin O., Rubio-Jurado B., Riebeling C., Nava A. (2014). The clinical significance of posttranslational modification of autoantigens. Clin. Rev. Allergy Immunol..

[88] Moulton V.R., Gillooly A.R., Tsokos G.C. (2014). Ubiquitination regulates expression of the serine/arginine-rich splicing factor 1 (SRSF_1_) in normal and systemic lupus erythematosus (SLE) T cells. J. Biol. Chem..

[89] Barrera-Vargas A., Gómez-Martín D., Carmona-Rivera C., Merayo-Chalico J., Torres-Ruiz J., Manna Z., Hasni S., Alcocer-Varela J. (2018). Differential ubiquitination in NETs regulates macrophage responses in systemic lupus erythematosus. Ann. Rheum. Dis..

[90] Quiroz E.N., Chavez-Estrada V., Macias-Ochoa K., Ayala-Navarro M.F., Flores-Aguilar A.S., Morales-Navarrete F., de la Cruz Lopez F., Escorcia L.G., Musso C.G., Martinez G.A. (2019). Epigenetic mechanisms and posttranslational modifications in systemic lupus erythematosus. Int. J. Mol. Sci..

[91] Wei J.-W., Huang K., Yang C., Kang C.-S. (2017). Non-coding RNAs as regulators in epigenetics (review). Oncol. Rep..

[92] Li J., Lui C. (2019). Coding or non-coding, the converging concepts of RNAs. Front. Genet..

[93] Turpin D., Truchetet M.-E., Faustin B., Augusto J.-F., Contin-Bordes C., Brisson A., Blanco P., Duffau P. (2016). Role of extracellular vesicles in autoimmune diseases. Autoimmun. Rev..

[94] Tan L., Wu H., Liu Y., Zhao M., Li D., Lu Q. (2016). Recent advances of exosomes in immune modulation and autoimmune diseases. Autoimmunity.

[95] Fan W., Liang D., Tang Y., Qu B., Cui H., Luo X., Huang X., Chen S., Higgs B.W., Jallal B. (2012). Identification of microRNA-31 as a novel regulator contributing to impaired inerleukin-2 production in T cells from patients with systemic lupus erythematosus. Arthritis Rheum..

[96] Lu M.-C., Lai N.-S., Chen H.-C., Yu H.-C., Huang K.-Y., Tung C.-H., Huang H.-B., Yu C.-L. (2013). Decreased microRNA (miR)-145 and increased miR-224 expression in T cells from patients with systemic lupus erythematosus involved in lupus immunopathogenesis. Clin. Exp. Immunol..

[97] Lu M.-C., Yu C.-L., Chen H.-C., Yu H.-C., Huang H.-B., Lai N.-S. (2015). Aberrant T cell expression of Ca^2+^ influx-regulated miRNAs in patients with systemic lupus erythematosus promotes lupus pathogenesis. Rheumatology.

[98] Khoshmirsafa M., Kianmehr N., Falak R., Mowla S.J., Seif F., Mirzaei B., Valizadeh M., Shekarabi M. (2019). Elevated expression of miR-21 and miR-155 in peripheral blood mononuclear cells as potential biomarkers for lupus nephritis. Int. J. Rheum. Dis..

[99] Li L.-J., Zhao W., Tao S.-S., Li J., Xu S.-Z., Wang J.-B., Leng R.-X., Fan Y.-G., Pan H.-F., Ye D.-Q. (2017). Comprehensive long non-coding RNA expression profiling reveals their potential roles in systemic lupus erythematosus. Cell. Immunol..

[100] Wang Y., Chen S., Chen S., Du J., Lin J., Qin H., Wang J., Liang J., Xu J. (2018). Long noncoding RNA expression profile and association with SLEDAI score in monocyte-derived dendritic cells from patients with systemic lupus erythematosus. Arthritis Res. Ther..

[101] Cao H.-Y., Li D., Wang Y.-P., Lu H.-X., Sun J., Li H.-B. (2020). Clinical significance of reduced expression of lncRNA TUG_1_ in the peripheral blood of systemic lupus erythematosus patients. Int. J. Rheum. Dis..

[102] Geng L., Xu X., Zhang H., Chen C., Hou Y., Yao G., Wang S., Wang D., Feng X., Sun L. (2020). Comprehensive expression profile of long non-coding RNAs in peripheral blood mononuclear cells from patients with neuropsychiatric systemic lupus erythematosus. Ann. Transl. Med..

[103] Gao F., Tan Y., Luo H. (2020). MALAT1 is involved in type I IFNs-mediated systemic lupus erythematosus by up-regulating OAS2, OAS3, and OASL. Braz. J. Med. Biol. Res..

[104] Ye H., Wang X., Wang L., Chu X., Hu X., Sun L., Jiang M., Wang H., Wang Z., Zhao H. (2019). Full high-throughput sequencing analysis of differences in expression profiles of long noncoding RNAs and their mechanisms of action in systemic lupus erythematosus. Arthritis Res. Ther..

[105] Gao X., Liu L., Min X., Jia S., Zhao M. (2020). Non-coding RNAs in CD4^+^ T cells: New insights into the pathogenesis of systemic lupus erythematosus. Front. Immunol..

[106] Perez-Hernandez J., Cortes R. (2015). Extracellular vesicles as biomarkers of systemic lupus erythematosus. Dis. Markers.

[107] Yáñez-Mό M., Siljander P.R.-M., Andreu Z., Zavec A.B., Borràs F.E., Buzas E.I., Buzas K., Casal E., Cappello F., Carvalho J. (2015). Biological properties of extracellular vesicles and their physiological functions. J. Extraceull. Vesicles.

[108] Valadi H., Ekström K., Bossios A., Sjöstrand M., Lee J.J., Lötvall J.O. (2007). Exosome-mediated transfer of mRNAs and microRNAs is a novel mechanism of genetic exchange between cells. Nat. Cell Biol..

[109] Perez-Hernandez J., Redon J., Cortes R. (2017). Extracellular vesicles as therapeutic agents in systemic lupus erythematosus. Int. J. Mol. Sci..

[110] Wang G., Tam L.-S., Li E.K.-M., Kwan B.C.-H., Chow K.-M., Luk C.C.-W., Li P.K.-T., Szeto C.-C. (2010). Serum and urinary cell-free miR-146a and miR-155 in patients with systemic lupus erythematosus. J. Rheumatol..

[111] Carlsen A.L., Schetter A.J., Nielsen C.T., Lood C., Knudsen S., Voss A., Harris C.C., Hellmark T., Segelmark M., Jacobsen S. (2013). Circulating microRNA expression profiles associated with systemic lupus erythematosus. Arthritis Rheum..

[112] Wang W., Mou S., Wang L., Zhang M., Shao X., Fang W., Lu R., Qi C., Fan Z., Cao Q. (2015). Up-regulation of serum miR-130b-3p level is associated with renal damage in early lupus nephritis. Sci. Rep..

[113] Amr K.S., Bayoumi F.S., Elgengehy F.T., Abdallah S.O., Ahmed H.H., Eissa E. (2016). The role of microRNA-31 and microRNA-21 as regulatory biomarkers in the activation of T lymphocytes of Egyptian lupus patients. Rheumatol. Int..

[114] Kay S.D., Carlsen A.L., Voss A., Burton M., Diederichsen A., Poulsen M.K., Heegaard N. (2019). Associations of circulating cell-free mcrioRNA with vasculopathy and vascular events in systemic lupus erythematosus patients. Scand. J. Rheumatol..

[115] Kim B.-S., Jung J.-Y., Jeon J.-Y., Kim H.-A., Suh C.-H. (2016). Circulating hsa-miR-30e-5p, hsa-miR-92a-3p, and hsa-miR-223-3p may be novel biomarkers in systemic lupus erythematosus. HLA.

[116] Zeng L., Wu J.-L., Liu L.-M., Jiang J.-Q., Wu H.-J., Zhao M., Lu Q.-J. (2018). Serum miRNA-371b-5p and miR-5100 act as biomarkers for systemic lupus erythematosus. Clin. Immunol..

[117] Navarro-Quiroz E., Pacheco-Lugo L., Navarro-Quiroz R., Lorenzi H., España-Puccini P., Díaz-Olmos Y., Almendrales L., Olave V., Gonzalez-Torres H., Diaz-Perez A. (2017). Profiling analysis of circulating microRNA in peripheral blood of patients with class IV lupus nephritis. PLoS ONE.

[118] Zhang H., Huang X., Ye L., Guo G., Li X., Chen C., Sun L., Li B., Chen N., Xue X. (2018). B cell related circulating microRNAs with the potential value of biomarkers in the differential diagnosis, and distinguishment between the disease activity and lupus nephritis for systemic lupus erythematosus. Front. Immunol..

[119] Zhang Y., Wang Y. (2018). The correlation of plasma microRNA-200 family expressions with risk and disease severity of lupus nephritis. Eur. Rev. Med. Pharmacol. Sci..

[120] Nakhjavani M., Etemadi J., Pourlak T., Mirhosaini Z., Vahed S.Z., Abediazar S. (2019). Plasma levels of miR-21, miR-150, miR-423 in patients with lupus nephritis. Iran. J. Kidney Dis..

[121] Abd-Elmawla M.A., Fawzy M.W., Rizk S.M., Shaheen A.A. (2018). Role of long non-coding RNAs expression (ANRIL, NOS_3_-AS, and APOA_1_-AS) in development of atherosclerosis in Egyptian systemic lupus erythematosus patients. Clin. Rheumatol..

[122] Wu G.-C., Hu Y., Guan S.-Y., Ye D.-Q., Pan H.-F. (2019). Differential plasma expression profiles of long non-coding RNAs reveal potential biomarkers for systemic lupus erythematosus. Biomolecules.

[123] Xu H., Chen W., Zheng F., Tang D., Liu D., Wang G., Xu Y., Yin L., Zhang X., Dai Y. (2020). Reconstruction and analysis of the aberrant lncRNA-miRNA-mRNA network in systemic lupus erythematosus. Lupus.

[124] Lucafò M., Di Silvestre A., Romano M., Avian A., Antonelli R., Martelossi S., Naviglio S., Tommasini A., Stocco G., Ventura A. (2018). Role of the long non-coding RNA growth arrest-specific 5 in glucocorticoid response in children with inflammatory bowel disease. Basic Clin. Pharmacol. Toxicol..

[125] Lucafò M., Pugnetti L., Bramuzzo M., Curci D., Di Silvestre A., Marcuzzi A., Bergamo A., Martelossi S., Villanacci V., Bozzola A. (2019). Long non-coding RNA GAS5 and intestinal MMP2 and MMP9 expression: A translational study in pediatric patients with IBD. Int. J. Mol. Sci..

[126] Wu J., Zhang T.-P., Zhao Y.-L., Li B.-Z., Leng R.-X., Pan H.-F., Ye D.-Q. (2020). Decreased H19, GAS5, and linc0597 expression and association analysis of related gene polymorphisms in rheumatoid arthritis. Biomolecules.

[127] Santoro M., Nociti V., Lucchini M., De Fino C., Losavio F.A., Mirabella M. (2016). Expression profile of long non-coding RNAs in serum of patients with multiple sclerosis. J. Mol. Neurosci..

[128] Ma J., Zhao N., Du L., Wang Y. (2019). Downregulation of lncRNA NEAT1 inhibits mouse mesangial cell proliferation, fibrosis, and inflammation but promotes apoptosis in diabetic nephropathy. Int. J. Clin. Exp. Pathol..

[129] Dong G., Yang Y., Li X., Yao X., Zhu Y., Zhang H., Wang H., Ma Q., Zhang J., Shi H. (2020). Granulocytic myeloid-derived suppressor cells contribute to IFN-I signaling activation of B cells and disease progression through the lncRNA NEAT1-BAFF axis in systemic lupus erythematosus. Biochim. Biophys. Act. Mol. Bas. Dis..

[130] Wang G., Tam L.S., Li E.K.M., Kwan B.C.H., Chow K.M., Luk C.C.W., Li P.K.T., Szeto C.C. (2011). Serum and urinary free microRNA level in patients with systemic lupus erythematosus. Lupus.

[131] Wang G., Tam L.-S., Kwan B.C.-H., Li E.K.-M., Chow K.-M., Luk C.C.-W., Li P.K.-T., Szeto C.-C. (2012). Expression of miR-146a and miR-155 in the urinary sediment of systemic lupus erythematosus. Clin. Rheumatol..

[132] Ichii O., Otsuka-Kanazawa S., Horino T., Kimura J., Nakamura T., Matsumoto M., Toi M., Kon Y. (2014). Decreased miR-26a expression correlates with the progression of podocyte injury in autoimmune glomerulonephritis. PLoS ONE.

[133] Perez-Hernandez J., Forner M.J., Pinto C., Chaves F.J., Cortes R., Redon J. (2015). Increased urinary exosomal microRNAs in patients with systemic lupus erythematosus. PLoS ONE.

[134] Solé C., Cortés-Hernández J., Felip M.L., Vidal M., Ordi-Ros J. (2015). miR-29c in urinary exosomes as predictor of early renal fibrosis in lupus nephritis. Nephrol. Dial. Transplant..

[135] Cardenas-Gonzalez M., Srivastava A., Pavkovic M., Bijol V., Rennke H.G., Stillman I.E., Zhang X., Parikh S., Rovin B.H., Afkarian M. (2017). Identification, confirmation, and replication of novel urinary microRNA biomarkers in lupus nephritis and diabetic nephropathy. Clin. Chem..

[136] Solé C., Moliné T., Vidal M., Ordi-Ros J., Cortés-Hernández J. (2019). An exosomal urinary miRNA signature for early diagnosis of renal fibrosis in lupus nephritis. Cells.

[137] Tangtanatakul P., Klinchanhom S., Sodsai P., Sutichet T., Promjeen C., Avihingsanon Y., Hirankarn N. (2019). Down-regulation of let-7a and miR-21 in urine exosomes from lupus nephritis patients during disease flare. Asian Pac. J. Allergy Immunol..

[138] Li Y.-J., Wu H.-H., Liu S.-H., Tu K.-H., Lee C.-C., Hsu H.-H., Chang M.-Y., Yu K.-H., Chen W., Tian Y.-C. (2019). Polyomavirus BK, BKV microRNA, and urinary neutrophil gelatinase-associated lipocalin can be used as potential biomarkers of lupus nephritis. PLoS ONE.

[139] Cao Y., Cao X., Sun L., Li Y. (2019). miR-206 inhibits cell proliferation and extracellular matrix accumulation by targeting hypoxia-inducible factor 1- alpha (HIF-1α) in mesangial cells treated with high glucose. Med. Sci. Monit..

[140] Garcia-Vives E., Solé C., Moliné T., Vidal M., Agraz I., Ordi-Ros J., Cortés-Hernández J. (2020). The urinary exosomal miRNA expression profile is predictive of clinical response in lupus nephritis. Int. J. Mol. Sci..

[141] Lu J., Kwan B.C.-H., Lai F.M.-M., Tam L.-S., Li E.K.-M., Chow K.-M., Wang G., Li P.K.-T., Szeto C.-C. (2012). Glomerular and tubulointerstitial miR-638, miR-198 and miR-146a expression in lupus nephritis. Nephrology.

[142] Zhou H., Hasni S.A., Perez P., Tandon M., Jang S.I., Zheng C., Kopp J.B., Austin H., Balow J.E., Alevizos I. (2013). miR-150 promotes renal fibrosis in lupus nephritis by downregulating SOCS1. J. Am. Soc. Nephrol..

[143] Krasoudaki E., Banos A., Stagakis E., Loupasakis K., Drakos E., Sinatkas V., Zampoulaki A., Papagianni A., Iliopoulos D., Boumpas D.T. (2016). Micro-RNA analysis of renal biopsies in human lupus nephritis demonstrates up-regulated miR-422a driving reduction of akllikrein-related peptidase 4. Nephrol. Dial. Transplant..

[144] Costa-Reis P., Russo P.A., Zhang Z., Colonna L., Maurer K., Gallucci S., Schulz S.W., Kiani A.N., Petri M., Sullivan K.E. (2015). The role of microRNAs and human epidermal growth factor receptor 2 in proliferative lupus nephritis. Arthritis Rheumatol..

[145] Yao F., Sun L., Fang W., Wang H., Yao D., Cui R., Xu J., Wang L., Wang X. (2016). Hsa-miR-371-5p inhibits human mesangial cell proliferation and promotes apoptosis in lupus nephritis by directly targeting hypoxia-inducible factor 1α. Mol. Med. Rep..

[146] Liu D., Zhang N., Zhang J., Zhao H., Wang X. (2016). miR-410 suppresses the expression of IL-6 as well as renal fibrosis in the pathogenesis of lupus nephritis. Clin. Exp. Pharmacol. Physiol..

[147] Leiss H., Salzberger W., Jacobs B., Gessl I., Kozakowski N., Blüml S., Puchner A., Kiss A., Podesser B.K., Smolen J.S. (2017). microRNA 155-deficiency leads to decreased autoantibody levels and reduced severity of nephritis and pneumonitis in pristane-induced lupus. PLoS ONE.

[148] Kong J., Li L., Lu Z., Song J., Yan J., Yang J., Gu Z., Da Z. (2019). microRNA-155 suppresses mesangial cell proliferation and TGF-β1 production via inhibiting CXCR5-ERK signaling pathway in lupus nephritis. Inflammation.

[149] Li X., Luo F., Li J., Luo C. (2019). miR-183 delivery attenuates murine lupus nephritis-related injuries via targeting mTOR. Scand. J. Immunol..

[150] Cui D., Zhu D., Ren H., Lin J., Lai W., Huang Q., Zhao J., Yang M. (2017). MciroRNA198 contributes to lupus nephritis progression by inhibition of phosphatase and tensin homology deleted on chromosome ten expression. Mol. Med. Rep..

[151] Zheng J., Guo R., Tang Y., Fu Q., Chen J., Wu L., Leng L., Bucala R., Song Y., Lu L. (2019). miR-152 attenuates the severity of lupus nephritis through the downregulation of macrophage migration inhibitory factor (MIF)-induced expression of COL1A1. Front. Immunol..

[152] Cai Z., Xiang W., Peng X., Ding Y., Liao W., He X. (2019). MicroRNA-145 involves in the pathogenesis of renal vascular lesions and may become a potential therapeutic target in patients with juvenile lupus nephritis. Kidney Blood Press. Res..

[153] Memczak S., Jens M., Elefsinioti A., Torti F., Krueger J., Rybak A., Maier L., Mackowiak S.D., Gregersen L.H., Munschauer M. (2013). Circular RNAs are a large class of animal RNAs with regulatory potency. Nature.

[154] Li P., Chen S., Chen H., Mo X., Li T., Shao Y., Xiao B., Guo J. (2015). Using circular RNA as a novel type of biomarker in the screening of gastric cancer. Clin. Chim. Acta.

[155] Huang G., Zhu H., Shi Y., Wu W., Cai H., Chen X. (2015). *cir-ITCH* plays an inhibitory role in colorectal cancer by regulating the Wnt/β–catenin pathway. PLoS ONE.

[156] Qin M., Liu G., Huo X., Tao X., Sun X., Ge Z., Yang J., Fan J., Liu L., Qin W. (2016). Has_circ_0001649: A circular RNA and potential novel biomarker for hepatocellular carcinoma. Cancer Biomark..

[157] Su H., Lin F., Deng X., Shen L., Fang Y., Fei Z., Zhao L., Zhang X., Pan H., Xie D. (2016). Profiling and bioinformatics analyses reveal differential circular RNA expression in radioresistant esophageal cancer cells. J. Trasl. Med..

[158] Li L.-J., Huang Q., Pan H.-F., Ye D.-Q. (2016). Circular RNAs and systemic lupus erythematosus. Exp. Cell Res..

[159] Li L.-J., Zhu Z.-W., Zhao W., Tao S.-S., Li B.-Z., Xu S.-Z., Wang J.-B., Zhang M.-Y., Wu J., Leng R.-X. (2018). Circular RNA expression profile and potential function of has_circ_0045272 in systemic lupus erythematosus. Immunology.

[160] Zhang C., Wang X., Chen Y., Wu Z., Zhang C., Shi W. (2018). The down-regulation of hsa_circ_0012919, the sponge for *miR-125a-3p*, contributes to DNA methylation of CD_11a_ and CD_70_ in CD4^+^ T cells of systemic lupus erythematosus. Clin. Sci..

[161] Quyang Q., Huang Q., Jiang Z., Zhao J., Shi G.-P., Yang M. (2018). Using plasma circRNA_002453 as a novel biomarker in the diagnosis of lupus nephritis. Mol. Immunol..

[162] Li H., Li K., Lai W., Li X., Wang H., Yang J., Chu S., Wang H., Kang C., Qui Y. (2018). Comprehensive circular RNA profiles in plasma reveals that circular RNAs can be used as novel biomarkers for systemic lupus erythematosus. Clin. Chim. Acta.

[163] Cortes R., Forner M.J. (2019). Circular RNAs: Novel biomarkers of disease activity in systemic lupus erythematosus?. Clin. Sci..

